# Localization of RNA and translation in the mammalian oocyte and embryo

**DOI:** 10.1371/journal.pone.0192544

**Published:** 2018-03-12

**Authors:** Denisa Jansova, Anna Tetkova, Marketa Koncicka, Michal Kubelka, Andrej Susor

**Affiliations:** 1 Institute of Animal Physiology and Genetics, CAS, Libechov, Czech Republic; 2 Department of Cell Biology, Faculty of Science, Charles University in Prague, Prague 2, Czech Republic; University of Toronto, CANADA

## Abstract

The tight correlation between mRNA distribution and subsequent protein localization and function indicate a major role for mRNA localization within the cell. RNA localization, followed by local translation, presents a mechanism for spatial and temporal gene expression regulation utilized by various cell types. However, little is known about mRNA localization and translation in the mammalian oocyte and early embryo. Importantly, fully-grown oocyte becomes transcriptionally inactive and only utilizes transcripts previously synthesized and stored during earlier development. We discovered an abundant RNA population in the oocyte and early embryo nucleus together with RNA binding proteins. We also characterized specific ribosomal proteins, which contribute to translation in the oocyte and embryo. By applying selected markers to mouse and human oocytes, we found that there might be a similar mechanism of RNA metabolism in both species. In conclusion, we visualized the localization of RNAs and translation machinery in the oocyte, that could shed light on this *terra incognita* of these unique cell types in mouse and human.

## Introduction

Meiotic maturation of mammalian oocytes and oocyte-to-zygote transition proceed without transcription and depend entirely on the post-transcriptional regulation of maternal mRNAs. The overall translation gradually decreases during oocyte meiotic maturation [1], but the activators of cap-dependent translation become more active during this period, implying a role for translation of specific mRNAs in the regulation of meiosis [2,3]. These mRNAs need to be recruited to translational machinery and subsequently degraded in a tightly controlled temporal manner [4]. Spatial segregation of protein synthesis in cells involves the positioning of mRNAs according to where their protein products are required, and results in local or compartmentalized gene expression. mRNA localization can occur during different stages of development. While these processes were studied in other cell types [5–7], little is known about mRNA localization and translation in the mammalian oocyte or early embryo.

Our previous study indicated that the oocyte nucleus contains an RNA population in the fully grown oocyte that most likely contributes to translation in the vicinity of chromosomes after nuclear envelope break down (NEBD) [8,9]. Localization of mRNAs at the spindle is evolutionarily conserved in mammals and has also been seen in mitotic cells [10,11]. The differences in enrichment of 384 mRNAs between the meiotic spindle area and cortical regions have been analyzed by microarrays [12]. Investigations of mRNA localization have been conducted in the oocytes of *Drosophila* [13,14], *Xenopus* [15,16] and mouse [4]. The localization of mRNA molecules within the cytoplasm provides a basis for cell polarization, underlying developmental processes such as asymmetric cell division during meiosis, and embryonic patterning [17].

Endogenous mRNAs do not exist alone; they bind to a number of proteins to form mRNA-protein complexes [18]. RNA-binding proteins (RBPs) and molecular motors mediate the transport of mRNAs along the cytoskeleton of cells, resulting in an asymmetric distribution of RNAs. RBPs are capable of regulating mRNA stability and translation. Some RBPs, such as CPE-binding protein (CPEB), Deleted in azoospermia-like (DAZL), post-transcriptionally regulate mRNA by binding to its 7-methylguanosine cap structure at the 5′-untranslated end (5′UTR) or to 3′-+ regions (3′UTRs). Regulation of the initiation of cap dependent translation is controlled via the translational repressor 4E-binding protein 1 (4E-BP1). Hierarchical phosphorylation of 4E-BP1 leads to its dissociation from eIF4E. In a previous study [19], we detected enriched localization of 4E-BP1 in the nucleus of fully grown oocytes. Another common mechanism regulating the recruitment and stability of dormant maternal mRNAs is reversible polyadenylation, which is controlled by cytoplasmic polyadenylation elements (CPEs) [20]. CPEs are specific sequences in 3′UTRs of dormant maternal mRNAs that serve as a binding platform for the CPE-binding proteins (CPEBs), which control polyadenylation-induced translation. The CPEBs family has four members. The most studied is CPE-binding protein 1 (CPEB1), which functions as a translational activator or repressor according to its phosphorylation state [21]. Other RBPs that regulate RNA processing are known as heterogeneous nuclear ribonucleoproteins (hnRNPs) [22]. This family contains more than 20 members. The key characteristic of the hnRNPs is their nucleocytoplasmic shuttling [23]. The hnRNP proteins A0, A1, A2/B1 and A3 were considered to be the prime constituents of 40S heterogeneous nuclear ribonucleoprotein particles, which bind to and stabilize nascent pre-mRNA [24,25]. The exon junction complex consists of RNA binding proteins and contains Eukaryotic initiation factor 4A-III (eIF4A3), a DEAD-box RNA helicase–a member of the eIF4A family of translation initiation factors [26]. Exon junction complex proteins play important roles in post-splicing events–including mRNA export, cytoplasmic localization, and nonsense-mediated decay [26,27,28]. The presence of assembled ribosomes is directly linked to protein synthesis during crucial periods of development and is tightly associated with the developmental competence of oocytes. It has been proposed that the mRNAs for 27 ribosomal proteins are expressed at higher levels in developmentally competent oocytes compared to non-competent ones [29].

A remarkable feature of mammalian oocyte maturation is the significant elimination of rRNA and ribosomes [30]. Moreover, genome-wide transcriptome analysis has shown that mRNAs coding for ribosomal proteins are degraded during oocyte maturation and after fertilization [31,32]. The eukaryotic ribosome contains 4 RNAs and ~80 ribosomal proteins (RPs), forming its two subunits: small 40S and large 60S. RPL24 is positioned between the two subunits with an N-terminal domain in 60S [33], while its C-terminal part interacts with RPS6 [34]. Mitogens and growth factors are responsible for RPS6 phosphorylation on five residues [35,36]. It was shown that RPS6 phosphorylation has an important function in the translational control of the subclass of mRNAs that harbor the 5′ tract oligopyrimidine (5′ TOP) sequence and this level of regulation may imbue the ribosome with greater specificity [37]. Similar to RPS6, RPS14 also plays a role in the regulation of the MDM2-p53 pathway [38,39].

In this study we focus on the visualization of transcriptome in association with the regulation of translation and visualization of translation in the mammalian oocyte and early embryo. Extensive analyses of the transcriptionally inactive germinal vesicle (GV) oocytes and the 2-cell embryos during transcriptional reprograming show substantial measurements of both similarities and differences between the two cell types, as well as between mouse and human. Here, we also present the distribution and expression of specific components of translational machinery in the mammalian oocyte and early embryo.

## Methods

### Oocyte isolation and cultivation

Mouse ovaries were obtained from CD1 mice which were at least 6 weeks old and had been stimulated to superovulate by intraperitoneal injection of 5 UI pregnant mare serum gonadotropin (PMSG; Folligon, Merck Animal Health) 46 h prior to collection. Growing and fully grown GV oocytes were isolated subsequently into M2 medium (Millipore) supplemented with 100 μM of 3-isobutyl-1-methylxanthine (IBMX, Sigma Aldrich) to prevent resumption of meiosis. Selected oocytes were stripped of cumulus cells and cultured in M16 medium (Millipore) without IBMX at 37°C, 5% CO_2_. Zygotes were obtained by mating females with males after injections of 5 UI PMSG and then (after 46 h) 5 UI of human chorionic gonadotropin (hCG; Merck Animal Health). Zygotes were isolated 17 h after mating and cultured *in vitro* in M16 under mineral oil for 20 h, then 2-cell embryos were collected. All animal work was conducted according to Act No 246/1992 on the protection of animals against cruelty. Human oocytes, not used in human reproduction, were obtained from the Obstetrics and Gynecology Clinic of the General University Hospital in Prague. The project was accredited (#30/12) by the Ethical Committee of the General Hospital, Prague.

### Immunocytochemistry

Oocytes were fixed 15 min in 4% paraformaldehyde (PFA, Sigma Aldrich) in PBS and permeabilized 10 min in 0.1% Triton X-100 in PBS with one drop of ActinGreen probe Phalloidin488 (Thermo Fisher). Then the oocytes were incubated overnight at 4°C with primary antibodies diluted in PBS/0.2% normal bovine serum. The following antibodies were used in 1:150 dilution: rabbit anti-4E-BP1 (CST); rabbit anti-Ribosomal S14 (Santa Cruz); rabbit anti-Ribosomal S3 (CST); rabbit anti-RPL24 (Thermo Fisher); mouse anti-RPS6 (Santa Cruz); rabbit anti-CPEB4 (Thermo Fisher); mouse anti-hnRNPA1 (Sigma Aldrich); mouse anti-eIF4A3 (Abcam), rabbit anti-RPL7 (Abcam); mouse anti-m3G cap/m7G cap (Thermo Fisher). Mouse anti-5.8S rRNA antibody (Abcam), was also diluted 1:150 and incubated at room temperature for 2 h. After washing in PBS for 2x15 min, detection of the primary antibodies was performed by cultivation of the oocytes with relevant Alexa Fluor 488/594 conjugates (diluted 1:250) for 1 h at room temperature. Oocytes were then washed 2x15 min in PBS and mounted using Vectashield Mounting Medium with DAPI (Vector Laboratories). For nascent protein synthesis oocytes and embryos were incubated 1 h with 1 μgml^-1^ puromycin (Sigma Aldrich) or 1 μM cycloheximide, specific stage (GV, 2-cell embryo). For measuring nascent translation oocytes were cultured in methionine-free medium (Gibco) supplemented with 1% dialyzed fetal bovine serum (10,000MW; Sigma) and 50 mM HPG for 30 min. HPG was detected by using Click-iT Cell Reaction Kit (Life Technologies) according manufacture instruction. Samples were visualized using Leica SP5 inverted confocal microscope in 16 bit depth. Images were assembled in Photoshop CS3 and quantified by Image J software (http://rsbweb.nih.gov/ij/). Experiments were performed three times with 25 oocytes/embryos per experiment.

### Preparation of slides for imaging

Nunc Lab-Tek II Chamber Slide System (Thermo Fisher) glass coverslips were coated at 37°C for 2 h with poly-L-ornithine (Sigma Aldrich) diluted in RNase-free water 1:250 and then overnight with laminin (Sigma Aldrich) diluted in PBS 1:1000 also at 37°C.

### RNA fluorescent in situ hybridization (FISH)

RNA FISH was performed with small changes according to [40]. Oocytes were fixed for 10 min in 4% paraformaldehyde and permeabilized in 0.1% Triton X-100 in PBS with 40 units/20 μl of RNAseOut (Invitrogen), then mounted to pre-coated Lab-Tek II Chamber Slide System slides using 80% methanol pre-frozen to -20°C. Oocytes were washed in Wash Buffer A (Biosearch Technologies) and incubated overnight at 30°C in hybridization buffer (Biosearch Technologies) with 75 nM oligo-d(T) probe (Biotech Generi); *Neat2* CalFluorRed610 (Biosearch Technologies); *Dazl* (Biosearch Technologies) and β-*actin* labelled with Cy5 (Biotech Generi); GFP CalFluorRed635 (Biosearch Technologies) with 75 nM (protected from light). Oocytes were then washed 3x in Wash Buffer A and 2x in 2xSSC (Sigma Aldrich). For visualization of chromatin structure the oocytes were incubated 1 min with 10 nM DAPI (Sigma Aldrich) in 2xSSC; then washed 1x with 2xSSC and scanned in 2xSSC. For negative control RNase A (Ambion) was used for 2 h at 37°C after the permeabilization step. Forty-five oocytes and embryos was analyzed using ImageJ/FIJI (http://rsbweb.nih.gov/ij/) for quantification of fluorescence intensity in the cytoplasm and the nucleus.

### Rolling circle amplification (RCA) FISH

RCA FISH was performed according to [41] protocol with the following changes: oocytes were fixed 10 min in 4% PFA (Sigma Aldrich) and permeabilized in 0.1% Triton X-100 in PBS for 10 min and subsequently in 70% ethanol, pre-frozen to -20°C, for 10 seconds. The whole transcriptome was converted into cDNA by M-MuLV reverse transcriptase (Enzymatics) and the reaction mix was prepared according to the mentioned protocol. The cDNA fragments were fixed to the cellular protein matrix using a nonreversible amine cross-linker BS(PEG)9 (Sigma Aldrich) and circularized after degrading the RNA residues. The circular templates were amplified using RCA primers 100 μM (TCTTCAGCGTTCCCGA*G*A; where * is phosphorothioate, Generi Biotech) complementary to the adapter sequence in the presence of aminoallyl-dUTP and stably cross-linked. For visualization of endogenous cDNA we used fluorescently labeled random octamers, which were labeled by two fluorophore (green-488 nm) or (red-561 nm). Chromatin was visualized by incubation (1min) with 10 nM DAPI (Sigma Aldrich) in 2xSSC; then washed 1x with 2xSSC. We scanned the samples in 2xSSC. Quantification of fluorescent intensity between cytoplasm and nucleus was used ImageJ/FIJI (http://rsbweb.nih.gov/ij/). Per one experiment was used 12 oocyte and 10 embryos, we quantified 3 experiments.

### *In situ* proximity ligation assay (PLA)

Proximity ligation assay was performed according to manual instructions of PLA Duolink kit (Sigma Aldrich). Oocytes and embryos were incubated 1 h with 1 μgml^-1^ puromycin (Sigma Aldrich) to decrease translation, then fixed 15 min in 4% paraformaldehyde in PBS and permeabilized 10 min in 0.1% Triton X-100 in PBS. We added PLA Duolink kit blocking solution to each sample. Oocytes were incubated with primary antibodies rabbit anti-RPL24 (Thermo Fisher) and mouse anti-RPS6 (Santa Cruz) at 4°C overnight. The samples were washed in PBS and then in Wash Buffer A (Sigma Aldrich). The samples were incubated with 40 μl reaction mixture (8 μl PLA probe MINUS stock, 8 μl PLA probe PLUS stock and 24 μl PBS) in a chamber for 1 h at 37°C. The slides were washed in 1x Wash Buffer A for 6x2 min and ligation was performed in 40 μl reaction: 1 μl of ligase to 39 μl of ligation solution. Samples were incubated in ligation reaction mixture for 30 min at 37°C then washed 6x2 min in Wash Buffer A. 40 μl of amplification reaction (0.5 μl polymerase and 39.5 μl amplification solution) was added to each sample before incubation at 37°C for 100 min. Next, the samples were washed in Wash Buffer B (Sigma Aldrich) for 3x5 min and in 0.01 Wash Buffer B for 2 min. The samples were mounted by Vectashield Mounting Medium with DAPI (Vector Laboratories). Quantification of interaction foci was performed using ImageJ/FIJI. We performed 5 experiments with 150 oocytes/embryos.

### Live cell imaging of nascent translation (ReAsH method)

The ReAsH method [42] was used to detect *in situ* translation. For the ReAsH we used plasmid #27123 (Adgene;[43]). The growing oocytes were injected according to the protocol by [44] with a plasmid diluted to ~40 ng/μl into nuclei. Oocytes were incubated overnight in 1μM cycloheximide (CHX; Sigma Aldrich) in M16 medium to prevent translation. After CHX wash oocytes were incubated for 30 min in M16 supplemented with ReAsH dye (final concentration 20 μM, Thermo Fisher) and then transferred into 250 μM 2,3-dimercaptopropanol (BAL buffer, Thermo Fisher) in M16 and immediately scanned on confocal microscope Leica TCS SP5. Thirty oocytes was quantified from three independent experiments.

### Western blot (WB)

Lysates of embryos and oocytes was analysed on 4–12% gradient acrylamide gel. Samples were transferred to polyvinyliden-fluoride membrane (Immobilon P; Merckmillipore) using blotting system (Biometra GmbH) at 5 mA/cm^2^ during 25 minute. Membranes were blocked for 1 h, at room temperature and then they were incubated at 4 ^ᵒ^C overnight with the following primary antibodies: rabbit anti-4E-BP1 (CST); rabbit anti-Ribosomal S14 (Santa Cruz); rabbit anti-Ribosomal S3 (CST); rabbit anti-RPL24 (Thermo Fisher); mouse anti-RPS6 S235/236 (Santa Cruz); rabbit anti-CPEB4 (Thermo Fisher); mouse anti-hnRNPA1 (Sigma Aldrich); mouse anti-eIF4A3 (Abcam), rabbit anti-RPL7 (Abcam), with 1% milk/TTBS (Tween-Tris-buffer saline; NaCl, Tween 20,2M; Tris pH 7,6; dH2O). After 3 cycles of 10 minute washing in 0,05% TTBS membrane was incubated at room temperature for 1 h in 3% milk with secondary antibody conjugated with peroxidase (Jackson Immunoresearch). Following washing step with 0,05% TTBS. Immunodetected proteins were visualized by ECL (Amersham, GE Healthecare Life Science) according instruction from supplier. Films were scanned using a GS-800 calibrated densitometer (Bio-Rad) and quantified 3 independent experiment for each protein for one experiment was used 100 oocytes/embryos, we normalized quantification to GAPDH, using ImageJ/FIJI (http://rsbweb.nih.gov/ij/).

### Statistical analysis

Mean and SD values were calculated using MS Excel, statistical significance of the differences between the groups was tested using Student’s t-test (PrismaGraph5) and P<0.05 was considered as statistically significant (marked by asterisk) *p<0.05; **p<0.01; ***p<0.001.

## Results

### Detection of global transcriptome in the oocyte and embryo

**Fig 1:**
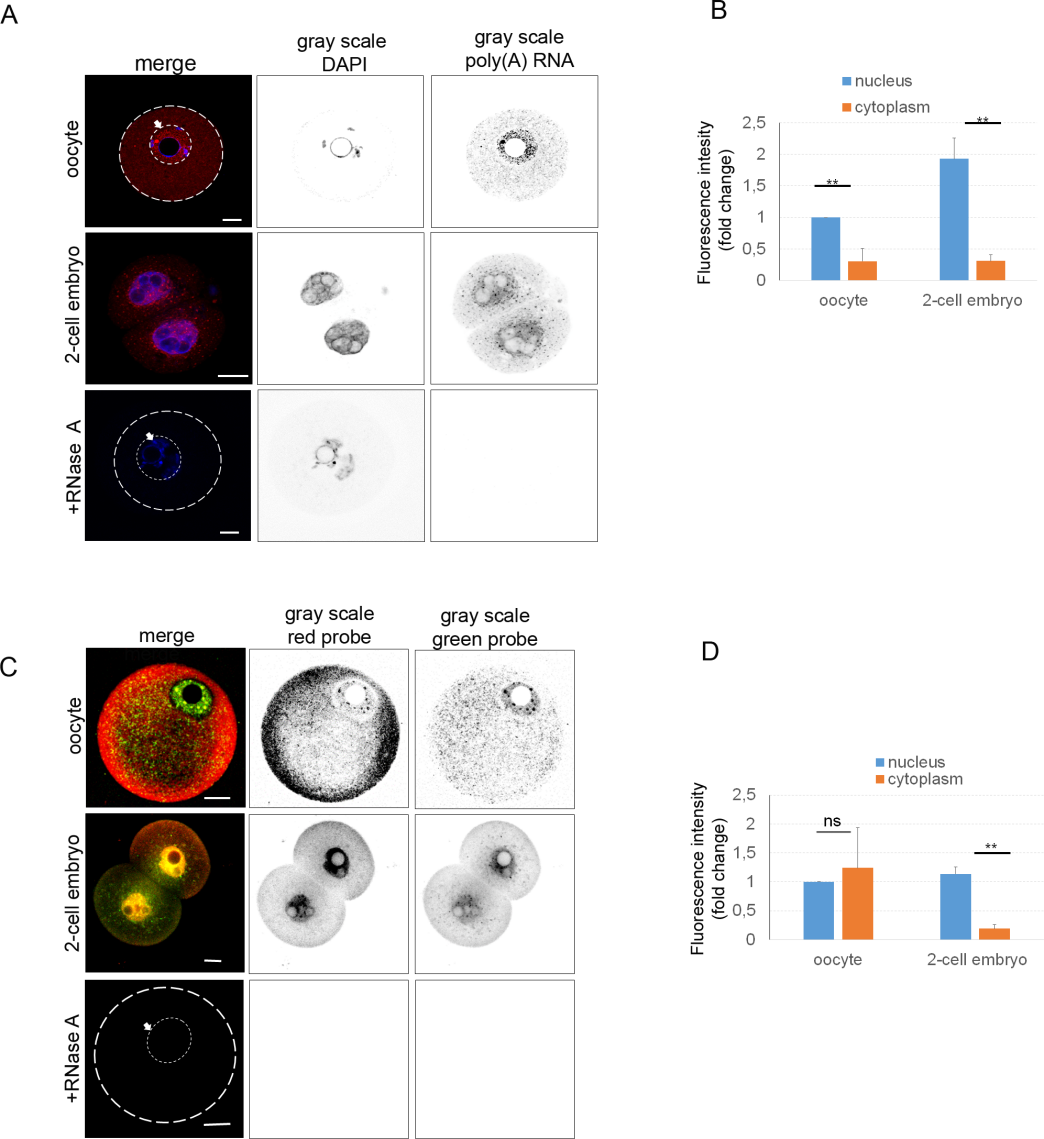
Localization of transcriptome in oocyte and embryo. A) Single Z-stack from confocal images of GV (germinal vesicle) oocyte stage and 2-cell embryo. RNA FISH detecting poly(A) RNA subpopulation (red; oligo(dT) probe), and the gray scale shows separated light channels (DAPI and oligo(dT) probe). The arrow with the white line indicates the nucleus of the oocyte. As a negative control RNA was digested by RNase A after the cell permeabilization step. Scale bars 20 μm. The cortex of the oocyte is indicated by the white line. B) Quantification of fluorescence intensity of poly(A) RNA of equatorial Z-stack, in the nucleus and cytoplasm of oocyte and embryo, relatively compared to the nucleus of the oocyte. The experiment was repeated 3 times, with 15 oocytes and embryos per experiment. Data are represented as mean ± s.d.; the values bars with *ns* are not significant, and the asterisk denotes statistically significant differences *p<0.05; **p<0.01; ***p<0.001. C) Rolling circle amplification FISH using random hexamers probes showing distribution of global RNA. The arrow with the white line indicates the nucleus of the oocyte. The gray scale shows separated channels (red and green probe). The cortex of the oocyte is indicated by the white line. As a negative control RNA was digested by RNase A after cell permeabilization step. Scale bars 20 μm. D) Quantification of fluorescence intensity of whole transcriptome in the nucleus and cytoplasm. The value of the nucleus was set as 1. The experiment was repeated 3 times, with 12 oocytes and 10 embryos per experiment. Data are represented as mean ± s.d.; the values bars with *ns* are not significant, and the asterisk denotes statistically significant differences *p<0.05; **p<0.01; ***p<0.001.

mRNA localization generally leads to targeted translation [45]. We have asked how the transcriptome is distributed in the mammalian oocyte and 2-cell embryo. It is known that 70% of RNAs are polyadenylated [46]. To detect this subpopulation, i.e. to detect RNA localization in the oocyte and 2-cell embryo, fluorescently (CY5) labeled oligo(dT) probes were used (Fig 1A). Treatment by RNase A eliminated the fluorescence signal, thus confirming its RNA origin in the rest of the experiments (Fig 1A). PolyA RNA was detected in the cytoplasm with a significant decrease (0.7-fold) compared to the nucleus of the oocyte. The nucleus of the 2-cell embryo shows higher (2-fold) presence of polyA RNA in the comparison with the oocyte. However, cytoplasmic signal intensity of poly(A) RNA is similar in both cell types (Fig 1B).

**Fig 2:**
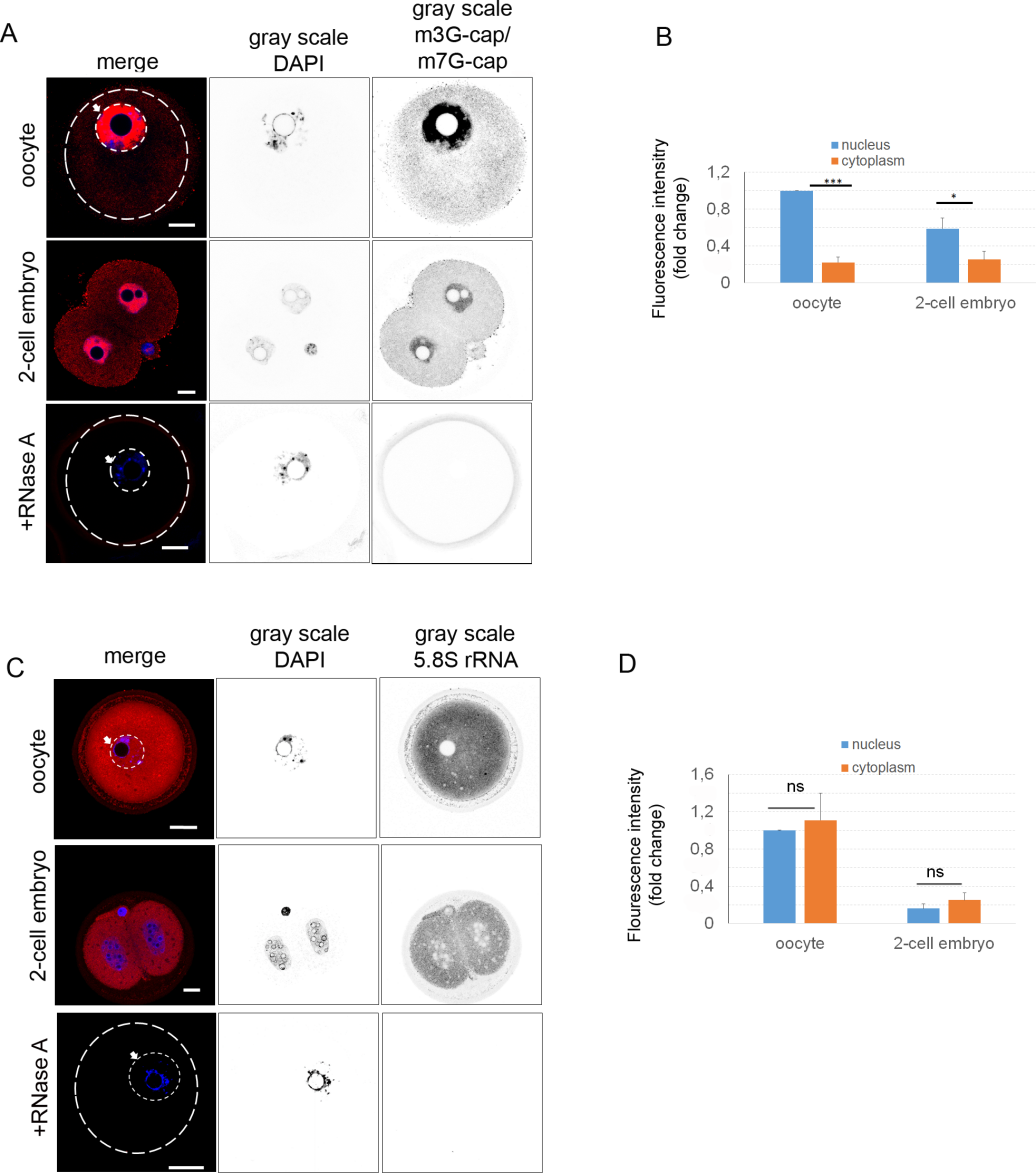
Localization of rRNA and RNA in oocyte and embryo. A) Antibody detecting m3G-cap and m7G-cap indicates cap-structure at the 5’UTR of mRNA (red). DNA stained with DAPI (blue). The gray scale shows separated light channels. The arrow with the white line indicates the nucleus of the oocyte. As a negative control RNA was digested by RNase A after the cell permeabilization step. The cortex of the oocyte is indicated by the white line. Scale bars 20 μm. The experiments were repeated 3 times, with 25 oocytes/embryos per experiment. B) Quantification of fluorescence intensity of 5’UTR cap-structure of equatorial Z-stacks, in the nucleus and cytoplasm of oocyte and embryo, relatively compared to nucleus of oocyte. The experiment was repeated 3 times, with 25 oocytes/embryos per experiment. Data are represented as mean ± s.d.; the value bars with *ns* are not significant, and the asterisk denotes statistically significant differences * p<0.05; **p<0.01; ***p<0.001. C) Distribution of 5.8S rRNA in the oocyte and early embryo (red). DNA stained with DAPI (blue). The gray scale shows separated light channels. The arrow with the white line indicates the nucleus of the oocyte. As a negative control RNase A digestion was used after the cell permeabilization step. The white line indicates the oocyte cortex. The experiment was repeated 3 times, with 25 oocytes/embryos per experiment. D) Quantification of fluorescence intensity of 5.8S rRNA in the nucleus and cytoplasm from equatorial Z-stacks. The value of the oocyte nucleus was set as 1; other values are represented as a ratio to the intensity of the oocyte nucleus. The experiment was repeated 3 times, with 25 oocytes/embryos per experiment. Data are represented as mean ± s.d.; the value bars with *ns* are not significant, and the asterisk denotes statistically significant differences * p<0.05; **p<0.01; ***p<0.001.

Rolling circle amplification (RCA) [41,47] was used to visualize the whole cellular transcriptome, the method comprising reverse *in situ* transcription followed by hybridization of fluorescently labeled random octamers, which were labeled by fluorophore, green (488 nm) and red (561 nm). By RCA, we observed a similar localization of RNA as with RNA FISH (Fig 1A and 1C). The different color pattern might be the result of a different affinity of the labeled probes. Similarly, the degradation of RNA by RNase A treatment significantly decreased the fluorescence signal (Fig 1C). Fluorescence intensity of global transcriptome was at the same level in both compartments of the oocyte (Fig 1D). However, in the cytoplasm of the 2-cell embryo RNA intensity was significantly lower (about 0.8-fold; Fig 1D). In addition to the previous methods for visualization of the global transcriptome, we used an antibody detecting the 5‘ UTR cap both m7G-cap (present in most RNAs) and m3G-cap (present in small nuclear RNAs, snRNAs) structures [48]. We were able to detect these cap structures (Fig 2A) with similar RNA distribution in both oocytes and 2-cell embryos, again comparable to RNA FISH and RCA methods (Fig 2A). Similar to RNA FISH and RCA results, we observed significantly higher fluorescence intensity of the m3G/m7G-cap in the nuclei of the oocytes and embryos (about 0.7-fold; Fig 2B).

Using RNA FISH, RCA and an antibody against RNA cap structure produced results showing a high uniformity concerning the localization of global transcriptome. We found the nuclei significantly enriched with RNA in both cell types.

In addition, using immunocytochemistry (ICC) with antibody against 5.8S ribosomal RNA, which is a structural component of 40S subunit of ribosome [49,50], we found a strong fluorescence signal in the cytoplasm of the oocyte and the embryo, with a decrease in the nucleoplasm of both stages (Fig 2C). Treatment with RNase A led to the loss of fluorescent signal. Quantification of 5.8S rRNA signal showed similar intensities in both cellular compartments in the oocyte/embryo. We observed a significant decrease of global 5.8S ribosomal RNA (about 0.8-fold) in the 2-cell embryo compared to the oocyte (Fig 2D).

Using direct detection of whole transcriptome by RCA, RNA FISH and ICC, we detected the localization of various RNA types and ribosomal rRNA in the mammalian oocyte and early embryo.

### Identification of specific RNAs in the oocyte and embryo

**Fig 3:**
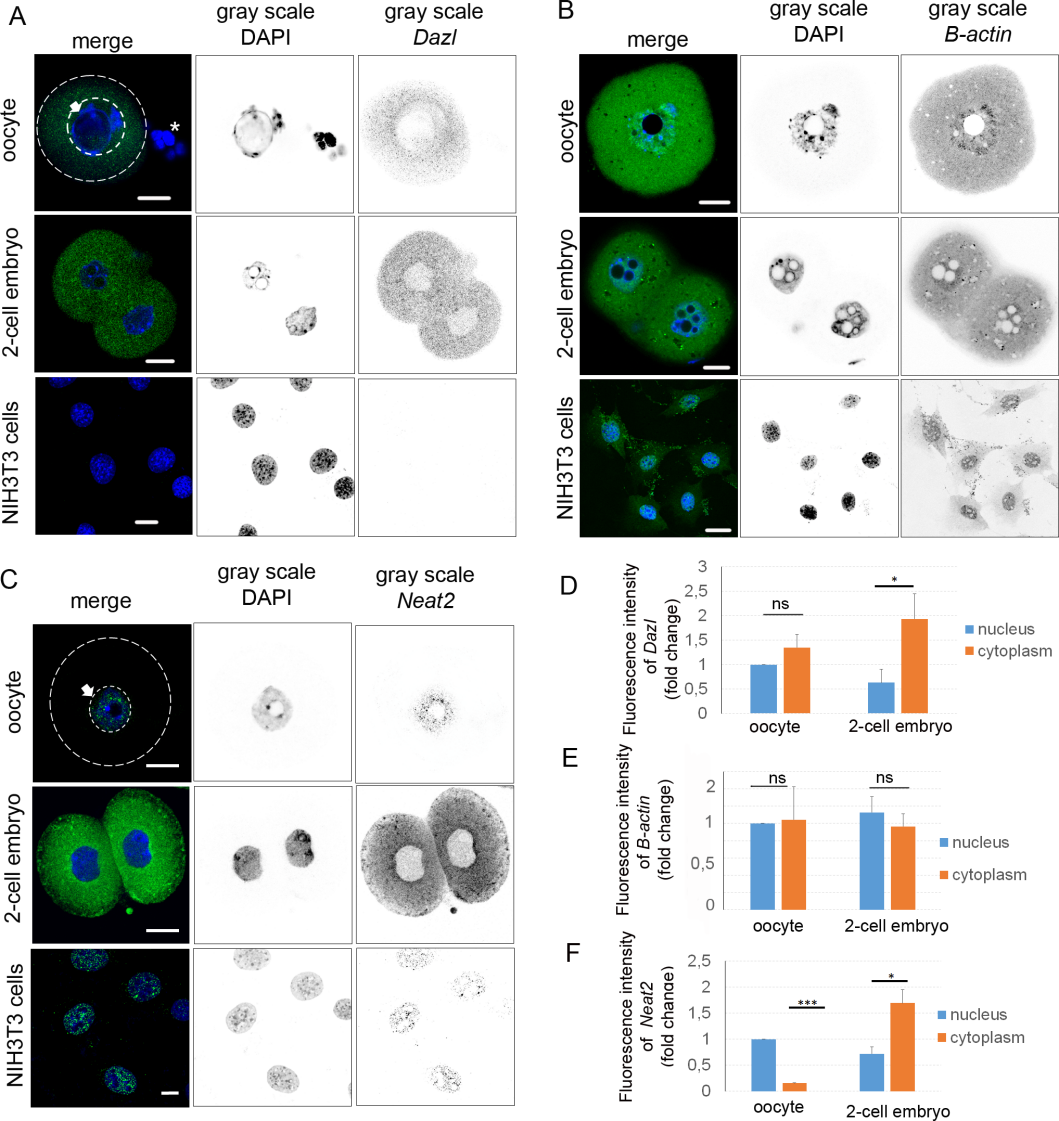
Localization of specific RNAs in GV oocyte, 2-cell embryo and NIH3T3 cells. A) Confocal images of smRNA FISH of *Dazl* (green) in GV oocyte, 2-cell embryo and NIH3T3 cells. DNA stained with DAPI (blue). The gray scale shows separated channels. The arrow with the white line indicates the nucleus of the oocyte. The asterisk indicates cumulus cells. The white line indicates the cortex of the oocyte. Scale bars 20 μm. The experiment was repeated 3 times, with 15 oocytes/embryos per experiment. B) Confocal images of smRNA FISH of β-*actin* (green) in GV oocyte, 2-cell embryo and NIH3T3 cells (DNA stained with DAPI (blue)). The gray scale shows separated light channels. Scale bars 20 μm. The experiment was repeated 3 times, with 15 oocytes and embryos per experiment. C) Confocal images of smRNA FISH of *Neat2* lncRNA (green) in GV oocyte, 2-cell embryo and NIH3T3 cells. DNA stained with DAPI (blue). The gray scale shows separated channels. The arrow with the white line indicates the nucleus of the oocyte. The white line indicates the cortex of the oocyte. Scale bars 20 μm. The experiment was repeated 3 times, with 15 oocytes/embryos per experiment. D) Quantification of fluorescence intensity of *Dazl mRNA* in the nucleus and cytoplasm from equatorial Z-stacks. The value of the oocyte nucleus was set as 1. The experiment was repeated 3 times, with 15 oocytes/embryos per experiment. Data are represented as mean ± s.d.; the values bars with *ns* are not significant, and the asterisk denotes statistically significant differences * p<0.05; **p<0.01; ***p<0.001. E) Quantification of fluorescence intensity of β-*actin* mRNA in the nucleus and cytoplasm from equatorial Z-stacks. Value of oocyte nucleus was set as 1. The experiment was repeated 3 times, with 15 oocytes and embryos per experiment. Data are represented as mean ± s.d.; the value bars with *ns* are not significant, asterisk denotes statistically significant differences * p<0.05; **p<0.01; ***p<0.001. F) Quantification of fluorescence intensity of *Neat2* lncRNA in the nucleus and cytoplasm from equatorial Z-stacks. The value of the oocyte nucleus was set as 1. The experiment was repeated 3 times, with 15 oocytes and embryos per experiment. Data are represented as mean ± s.d.; the value bars with *ns* are not significant, and the asterisk denotes statistically significant differences * p<0.05; **p<0.01; ***p<0.001.

Pinpointing the subcellular localization of specific RNA species might lead to the unveiling of their potential molecular role in such large cells. Single molecule RNA FISH (smRNA FISH) was used to visualize specific RNAs. First, we determined the localization of Deleted in azoospermia-like RNA (*Dazl*)–a germ cell specific transcript. smRNA FISH showed an even distribution of the mRNA in the cytoplasm and a weak signal in the nucleoplasm of both, the oocyte and the early embryo (Fig 3A and 3D) with increased levels in the cytoplasm of the 2-cell embryo (Fig 3A and 3D), suggesting early transcription of *Dazl* mRNA in the embryo. Cumulus cells (depicted by asterisk, Fig 3A) and mouse fibroblasts NIH3T3 (Fig 3A) were negative and RNase A treatment of the oocyte and embryo resulted in the absence of a fluorescent signal (S1B Fig), supporting the specificity of *Dazl* mRNA detection in the oocyte and early embryo. Another detected mRNA was β-*actin*, which was present in both subcellular compartments, nucleus and cytoplasm of the oocyte, and as dot-like structures in the cytoplasm of the early embryo (Fig 3B and 3E). The localization in the NIH3T3 was mostly at the leading edges (Fig 3B). Next, we also detected long noncoding RNA *Neat2/Malat1* (Nuclear-Enriched Abundant Transcript 2/Metastasis Associated Lung Adenocarcinoma Transcript 1) which is known to be localized in the nuclear speckles of HeLa cells [51]. *Neat2/Malat1* was exclusively localized in the nucleus of the GV oocyte and NIH3T3 cells. However, localized in the cytoplasm of the embryo (Fig 3C and 3F). RNase A treatment eliminated the fluorescent signal (S1B Fig) which corroborates the specificity of our RNA detection. The quantification of the intensity of the specific RNA signal shows that *Dazl* mRNA is distributed in both compartments, i.e. the nucleus and cytoplasm, of the oocyte (Fig 3D). However, in the embryo, this mRNA is elevated and localized predominantly in the cytoplasm (1.3-fold; Fig 3D). β-*actin* mRNA shows equal localization in the nucleus and embryo in both cell types (Fig 3E). *Neat2* lncRNA shows significant presence in the nucleus (about 0.85-fold) of the oocyte. In contrast, in the embryo, the fluorescence intensity was significantly higher (about 0.7-fold) in the cytoplasm (Fig 3F).

By detection of specific RNAs, we determined the localization of specific mRNAs and lncRNA in the oocytes, 2-cell embryos and mouse embryonic fibroblasts.

### Expression and localization of RNA binding proteins in the oocyte and 2-cell embryo

**Fig 4:**
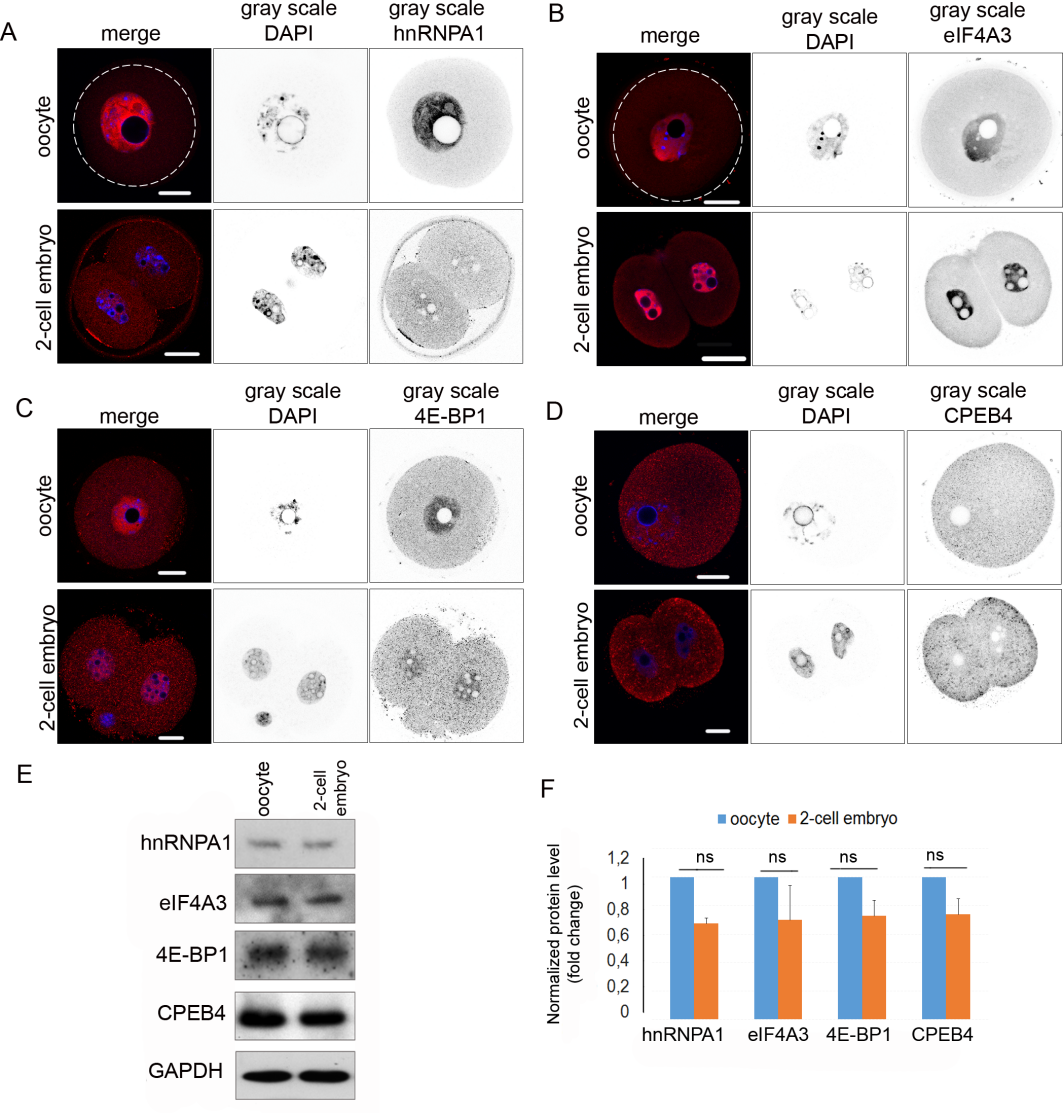
Localization and expression levels of RNA binding proteins in GV oocyte and 2-cell embryo. A) Representative confocal images of GV oocyte and 2-cell stage embryo stained with hnRNPA1 antibody (red), DNA stained with DAPI (blue); the gray scale images show separated light channels. The white line indicates the cortex of the oocyte. Scale bars 20 μm. The experiment was repeated 3 times, with 25 oocytes/embryos per experiment. B) Representative confocal image of GV oocyte and 2-cell embryo stained with eIF4A3 antibody (red), DNA stained with DAPI (blue); the gray scale images show separated light channels. The white line indicates the cortex of the oocyte. Scale bars 20 μm. The experiment was repeated 3 times, with 25 oocytes/embryos per experiment. C) Representative confocal image of GV oocyte and 2-cell embryo stained with 4E-BP1 antibody (red), DNA stained with DAPI (blue); the gray scale images show separated light channels. White line indicate cortex of oocyte. Scale bars 20 μm. The experiment was repeated 3 times, with 25 oocytes per experiment. D) Representative confocal image of GV oocytes and 2-cell embryo stained with CPEB4 antibody (red), DNA stained with DAPI (blue); the gray scale images show separated light channels. The white line indicates the cortex of the oocyte. Scale bars 20 μm. The experiment was repeated 3 times, with 25 oocytes/embryos per experiment. E) Representative images from WB probed by antibodies for hnRNPA1, eIF4A3, 4E-BP1, and CPEB4 proteins in the GV oocytes and 2-cell stage embryos. GAPDH was used as a loading control. WBs were performed 3 times, with 100 cells per experiment. F) Quantification of hnRNPA1, eIF4A3, 4E-BP1 and CPEB4 expression in the oocytes and embryos. Data were normalized to GAPDH. The values of oocytes were set as 1. Data are represented as mean ± s.d.; the value bars with *ns* are not significant, and the asterisk denotes statistically significant differences * p<0.05; **p<0.01; ***p<0.001.

RBPs are essential for RNA metabolism and localization [52–54], and so we accordingly examined the localization of a number of these proteins in the oocytes and early embryos. First, we studied the ubiquitously binding RBPs, heterogeneous nuclear ribonucleoproteins (hnRNP), which participate in pre-mRNA processing and are important determinants of mRNA export localization, translation, and stability [55]. Heterogeneous nuclear ribonucleoprotein A1 (hnRNPA1) was localized mostly in the nucleus of the oocyte (Fig 4A). However, in the embryo, the protein was present throughout the whole volume of the cell (Fig 4A). Next, we analyzed the distribution of the exon junction complex protein eIF4A3, which is deposited onto mRNA during splicing and is released during the first round of translation [26,56]. ICC showed a high abundance of eIF4A3 in the nuclei of both cell types, with a weak presence in the cytoplasm (Fig 4B). Another protein, 5’UTR cap-binding protein 4E-BP1, functions as a repressor of cap dependent translation [19,57,58]. In our experiments, 4E-BP1 showed a granular structure in the cytoplasm with a significant increase of signal in the nucleoplasm (Fig 4C and S2 Fig). RNase A treatment disrupted the granular pattern in the oocyte (S2 Fig). CPEB4 is responsible for meiotic progression between MI and MII and for the regulation of cytostatic factors in the frog oocyte [59]. CPEB4 analysis showed a granular distribution in the whole volume of the cells (Fig 4D). Next, WB was used to validate the antibodies (Fig 4E and 4F and S3 Fig). Expression levels of the hnRNPA1, eIF4A3, 4E-BP1 and CPEB4 showed no significant decrease in the early embryo (Fig 4F). The GAPDH protein was used as a loading control for WB.

### Localization of translational machinery

**Fig 5:**
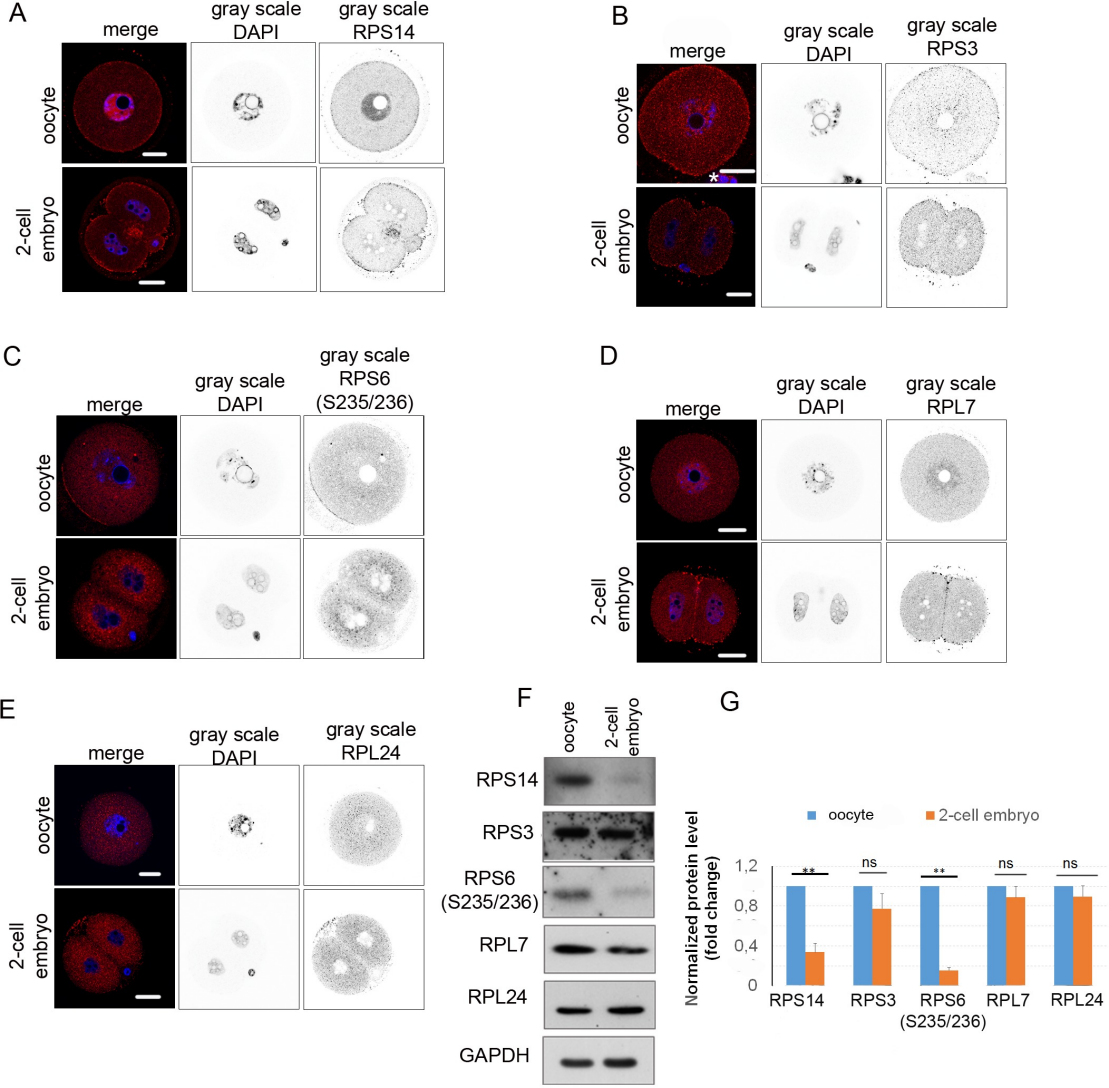
Localization and expression levels of selected ribosomal proteins. A) Confocal images of GV oocyte and 2-cell stage embryo probed with RPS14 antibody (red), DNA stained with DAPI (blue); the gray scale shows separated light channels. Scale bars 20 μm. The experiment was repeated 3 times, with 25 oocytes/embryos per experiment. B) Confocal images of GV oocyte and 2-cell stage embryo probed with RPS3 antibody (red), DNA stained with DAPI (blue); the gray scale shows separated light channels. The asterisk indicates cumulus cells. Scale bars 20 μm. The experiment was repeated 3 times, with 25 oocytes/embryos per experiment. C) Confocal images of GV oocyte and 2-cell stage embryo probed with RPS6(S235/236) antibody (red), DNA stained with DAPI (blue); the gray scale shows separated light channels. Scale bars 20 μm. The experiment was repeated 3 times, with 25 oocytes/embryos per experiment. D) Confocal images of GV oocyte and 2-cell stage embryo probed with RPL7 antibody (red); DNA stained with DAPI (blue); the gray scale shows separated light channels. Scale bars 20 μm. The experiment was repeated 3 times, with 25 oocytes/embryos per experiment. E) Confocal images of GV oocyte and 2-cell stage embryo probed with RPL24 antibody (red). DNA stained with DAPI (blue); the gray scale images shows separated light channels. Scale bars 20 μm. The experiment was repeated 3 times, with 25 oocytes/embryos per experiment. F) Representative images from WBs for RPS14, RPS3, RPS6 (S235/236), RPL7, RPL24 protein expression in GV oocytes and 2-cell stage embryos, and the loading control (GAPDH); WB was repeated 3 times, with 100 oocytes/embryos per experiment. G) Quantification of expression of RPS14, RPS3, RPS6 (S235/236), RPL7 and RPL24 proteins in the oocyte and embryo. Data were normalized to GAPDH. Data are represented as the mean ± s.d.; the values obtained from oocytes were set as 1. The value bars with *ns* are not significant, the asterisk denotes statistically significant differences *p<0.05; **p<0.01; ***p<0.001.

To characterize the localization and expression of ribosomal proteins in the GV oocyte and 2-cell embryo, we selected several components of the 40S ribosomal subunit–ribosomal proteins S14 (RPS14) and S3 (RPS3) and RPS6 phosphorylated on S235/236 –as well as of the 60S subunit, ribosomal protein L7 (RPL7) and L24 (RPL24). These subunits combine to form the eukaryotic (80S) ribosome [33], a large molecular machine that catalyzes the synthesis of proteins. ICC analysis showed that RPS14 was localized at the cortex of the oocyte and embryo and in the nucleoplasm of the oocyte (Fig 5A). However, in the 2-cell embryo we found a significant decrease of fluorescence in the nucleoplasm and cytoplasm, which was also confirmed by WB analysis (Fig 5A, 5F and 5G). The RPS3 protein was localized evenly in both compartments–nucleus and cytoplasm in the oocyte and the embryo (Fig 5B). The protein level of RPS3 was stable in the oocyte and 2-cell embryo (Fig 5F and 5G). It is known that phosphorylation of RPS6 on S235/236 increases its affinity to the cap structure of RNA, which strongly implies that RPS6 phosphorylation enhances mRNA translation initiation [60]. RPS6 protein phosphorylated on S235/236 was distributed throughout the whole cytoplasm with a decreased signal in the nucleoplasm in both the oocyte and the embryo (Fig 5C). RPS6(S235/236) significantly decreased its presence in the 2-cell embryo (Fig 5C, 5F and 5G). Protein RPL7 was distributed in the cytoplasm of the oocyte and embryo and with higher intensity in the nucleus of the oocyte (Fig 5D) compared to the embryo. Protein levels of RPL7 were stable in the oocyte and 2-cell embryo (Fig 5F and 5G). RPL24 showed more abundant distribution in the cytoplasm than in the nucleus in both cell types (Fig 5E). The quantification of the expression of ribosome components by WB in the oocyte and the embryo showed a significant decrease only for RPS14 (0.67-fold) and RPS6 phosphorylated on S235/236 (0.85-fold) in the 2-cell embryos (Fig 5F and 5G). Other components of ribosome, RPS3, RPL7 and RPL24 showed no significant decrease in the embryo (Fig 5F and 5G). The GAPDH protein was used as a loading control.

We showed different localization and expression of components of 40S and 60S subunits in the mouse oocyte and early embryo.

### Nascent translation in oocyte and 2-cell embryo

**Fig 6:**
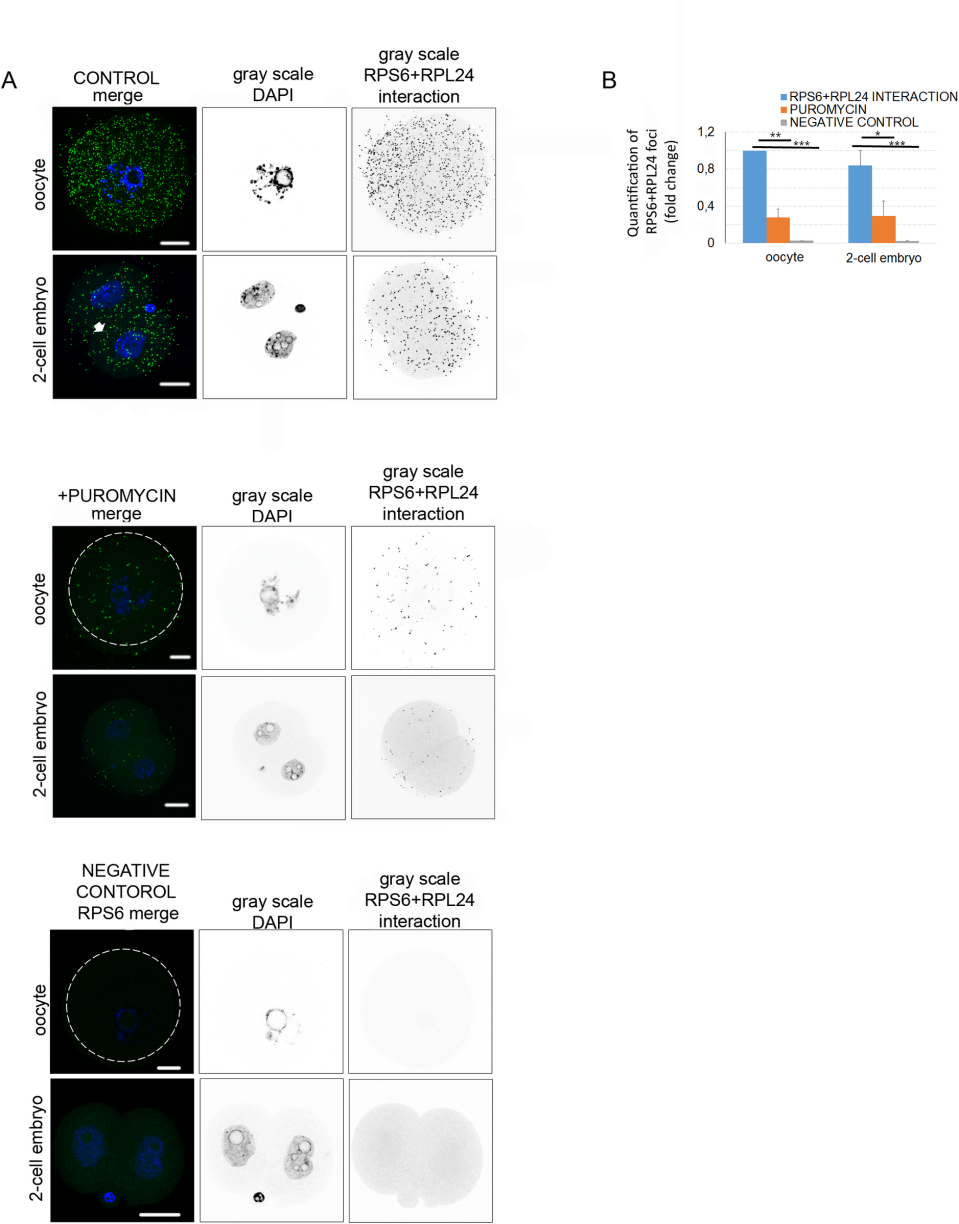
Detection of in situ translation in oocyte and embryo. A) Proximity ligation assay (PLA) detects interaction of two translational components RPL24 and RPS6 in the oocyte and 2-cell embryo. Fluorescent foci indicate RPL24 and RPS6 interactions (green) in the oocyte and embryo. As negative controls, oocytes and 2-cell embryos were treated with the translational inhibitor puromycin, or a single RPS6 antibody was used. DNA stained with DAPI (blue); the gray scale shows separated channels. Scale bar 20μm. The experiment was repeated 3 times, with 25 oocytes/embryos per experiment. B) Graph shows quantification of RPL24 and RPS6 interactions in the whole cell volume. The values obtained from the oocyte were set as 1. The experiment was repeated 3 times, with 25 oocytes/embryos per experiment. Data are represented as mean ± s.d.; the value bars with *ns* are not significant, and the asterisk denotes statistically significant differences *p<0.05; **p<0.01; ***p<0.001.

In order to detect ongoing protein synthesis, we used the Duolink *in situ* proximity ligation assay (PLA) [61,62] with RPL24 and RPS6 specific antibodies to detect assembled 80S ribosomes in the oocyte and embryo. The positive interaction of the RPL24 and RPS6 ribosomal proteins suggests completion of 80S ribosome and ongoing translation. Through PLA, we detected significant interactions of these two proteins in the cytoplasm of the oocyte and 2-cell embryo (Fig 6A). The quantification of the RPL24 and RPS6 interaction foci showed a similar number of RPL24 and RPS6 interactions in the oocyte and the embryo (Fig 6B). The distribution of interactions was even, with increased intensity at the cortex of blastomeres. The cultivation of oocytes or embryos in the presence of translational inhibitor puromycin (which disrupts translating ribosomes) showed a significant decrease (0.7-fold) in the number of interactions in both cell types (Fig 6A and 6B). Moreover, using RPS6 antibody alone as a negative control of PLA showed 0.98-fold significant decrease (Fig 6A and 6B). In order to detect nascent translation *in situ*, the methionine analog L-homoproparglycine (HPG) was incorporated into the translated proteins during a short 30-minute cultivation period followed by the Click-IT protocol to fluorescently label HPG in the cell [63]. The fluorescent signals of HPG were observed in the whole oocyte, with an increase in the perinuclear area (S4A Fig), while in the 2-cell embryo we detected strong signals at the dividing ridge of the blastomeres. As expected, the disruption of the ribosomes by puromycin decreased the intensity of the HPG signal in the oocyte by about 95%, and in the 2-cell embryo by about 80% (S4A and S4B Fig). In the presence of cycloheximide (CHX; protein elongation inhibitor) the intensity of the HPG signal in the oocyte and the embryo decreased to 15% and 13% respectively (S4A and S4B Fig).

The combination of PLA and HPG methods provided consistent visualization of the *in situ* translation in the oocyte and 2-cell embryo.

### Visualization of translation of endogenous β-*actin* mRNA in live oocyte

**Fig 7:**
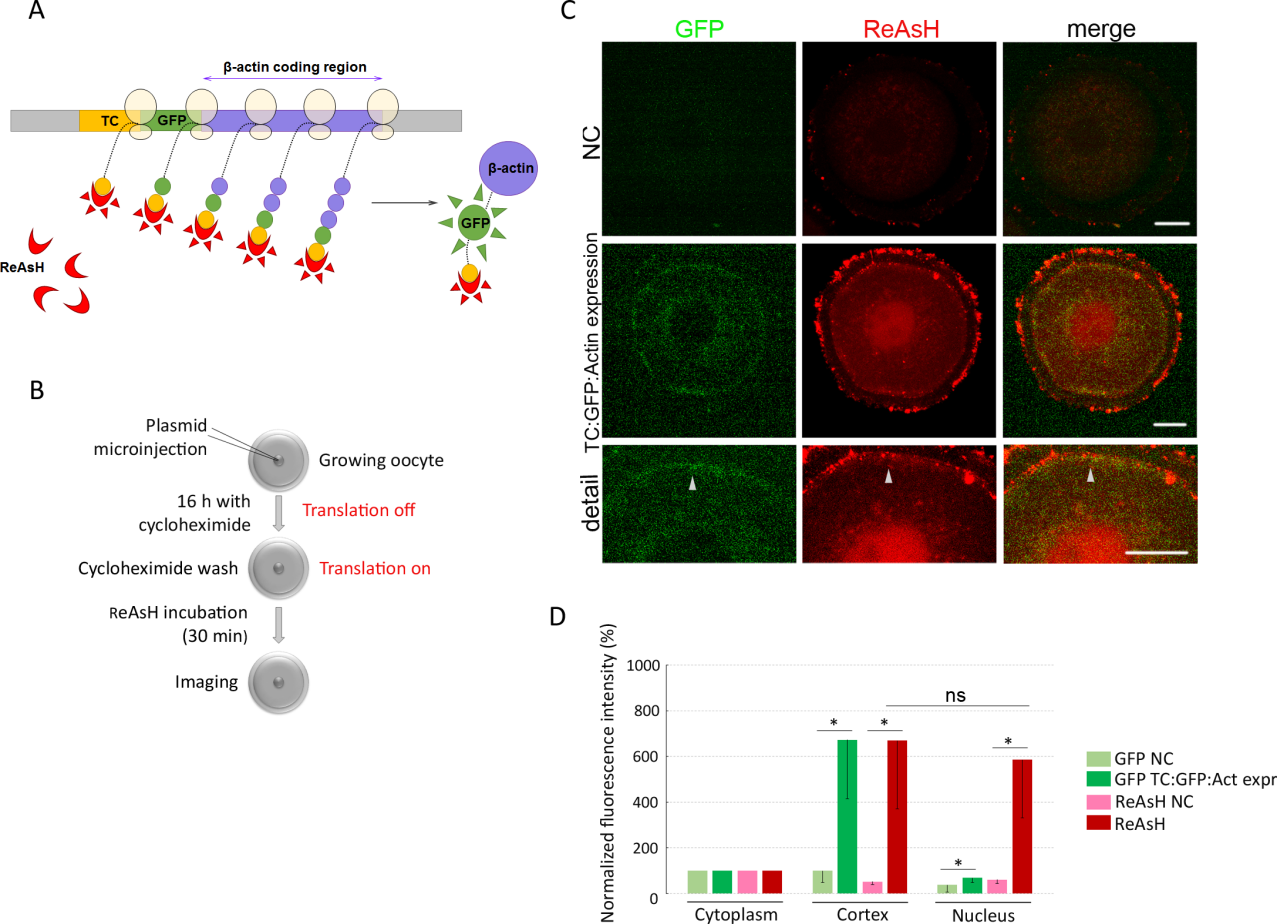
Visualization of translation of endogenous β-actin mRNA in live oocyte. A) Scheme of the TC GFP *β*-*actin* construct and detection of nascent translation. TC—tetracystein sequence; GFP—green fluorescent protein sequence; β-actin—open reading frame; ReAsH—resorufin arsenical hairpin binder. B) Scheme of the experimental procedures for translational detection of the *β*-*actin* RNA in the live oocyte. C) Representative confocal images of the oocyte microinjected with TC GFP β-actin plasmid and of non-injected control. ReAsH dye labels TC tag (red) of newly translated β-actin protein. GFP (green) was used as a marker of fully translated *β-actin*. The arrowheads depict nascent translation of β*-actin* RNA. Scale bars 10 µm. D) Quantification of the fluorescence intensity in the cytoplasm, cortex and nucleus. The values of the cytoplasm were set as 100%. The experiment was repeated 3 times, with 10 oocytes per experiment. Data are represented as mean ± s.d.; the value bars with *ns* are not significant; and the asterisk denotes statistically significant differences *p<0.05; **p<0.01; ***p<0.001.

The ReAsH method [42] was used to detect *in situ* translation of the specific transcript *β-actin* (Fig 7A). The plasmid coding the sequence of the β-actin ORF with tetracysteine (TC) and GFP domain [43] was injected into the nucleus of transcriptionally active oocytes in the presence of cycloheximide (CHX, protein synthesis inhibitor) (Fig 7B). Overnight cultivation of oocytes led to the transcription of the construct, which mimicked the endogenous *β-actin* mRNA. We found translation of β-actin in patches at the cortex of the oocyte, where ReAsH and GFP fluorescence were localized. GFP visualized mature protein on the cortex and ReAsH showed spots of *in situ* translation of *β-actin* (Fig 7C). A quantification of the images showed a sevenfold increase of the fluorescent intensity in ReAsH and GFP in the patches in the cortex (Fig 7D) compared with other areas of the cell or with negative control (non-injected oocytes). However, we detected a high presence of the ReAsH dye in the nucleus as well, where GFP was not detected (Fig 7C), which might suggest non-specific incorporation or an unknown process in the oocyte [64].

We were able to detect translation of the endogenous *β*-*actin* mRNA in the living oocyte, which resembled the known localization of filamentous actin [65,66], shown also in S5 Fig.

### Localization of global RNA and RBPs in the human oocyte

**Fig 8:**
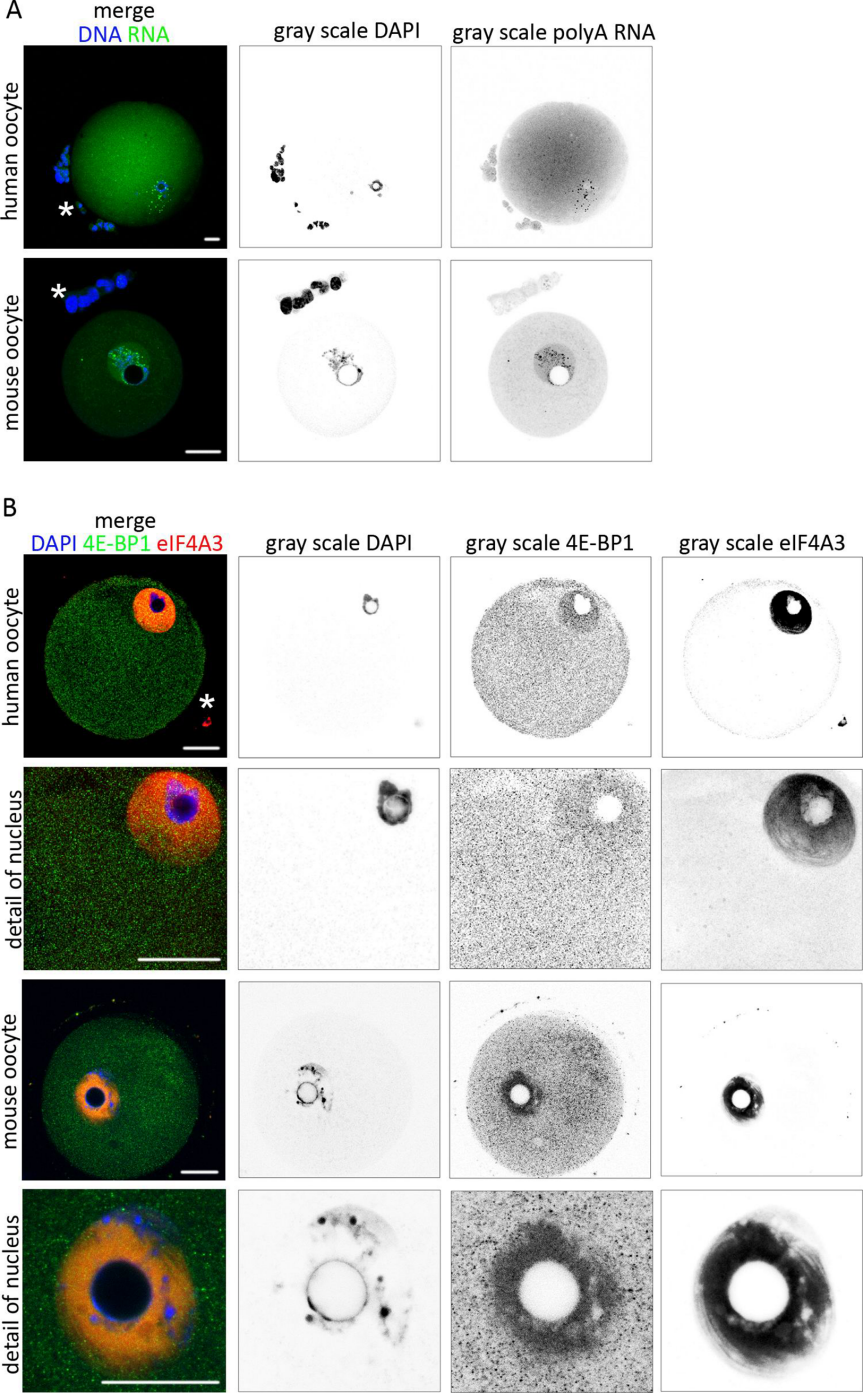
Localization of poly(A) RNA and RNA binding proteins in human oocyte. A) Single Z-stack from confocal images of human and mouse GV oocytes. RNA FISH detecting poly(A) RNA subpopulation (green) and DNA (blue). The gray scale shows separated light channels. The asterisk indicates cumulus cells. The experiment was repeated 3 times, with 15 mouse oocytes per experiment and 3 human oocytes per experiment. Scale bars 20 μm. B) Single Z-stack of human and mouse GV oocytes and 2-cell stage mouse embryos probed with 4E-BP1 (green) and eIF4A3 (red) and DAPI (blue). The gray scale shows separated channels. The experiment was repeated 3 times, with 15 mouse oocytes and 3 human oocytes. Scale bars 20 μm. The asterisk indicates cumulus cells.

In order to study the similarity between mouse and human oocytes, a localization of the global transcriptome was determined through the visualization of the poly(A) RNA population. We found that the poly(A) RNA fluorescence signal was distributed evenly throughout the cytoplasm and nucleoplasm with the presence of the abundant poly(A) RNA foci in the cytoplasm and nucleus of the human oocyte (Fig 8A). Next, the ICC labeling of RBPs, 4E-BP1 (green) and eIF4A3 (red) was performed, uncovering a similar localization of these proteins in human oocytes to our previously reported findings in the mouse oocyte [19] (Fig 8B). 4E-BP1 was distributed in both, the cytoplasm and the nucleoplasm. Contrastingly, eIF4A3 was localized in the nucleoplasm only (Fig 8B). Through analyses of the poly(A) RNA population and RBPs, we found similar localization of the selected transcriptome markers in both mammalian species.

## Discussion

Post-transcriptional control of gene expression at the level of translation has been shown to be essential for the regulation of a number of cellular processes during development [19,67]. Mammalian oocytes are transcriptionally active during their growth period. However, after resumption of meiosis, the oocytes reach a phase of transcriptional quiescence and utilize a store of maternally synthesized RNAs. The progression through meiosis and early embryogenesis is therefore regulated in the oocyte at the level of mRNA stabilization, translation and post-translational modifications. The aim of this study was to detect the global transcriptome present in the mammalian GV oocyte and the 2-cell embryo as a prerequisite for protein synthesis. Using RCA, poly(A) and RNA antibody detection techniques, we observed RNA distribution in both the cytoplasm and the nucleus of transcriptionally inactive oocytes, as well as in active 2-cell stage embryos, consistent with previous analyses [1,8]. Our results lead us to the conclusion that the transcriptionally inactive nucleus might serve as a repository for translationally dormant mRNAs, which may later be translated when the nuclear envelope breaks down after the resumption of meiosis. However, the presence of RNAs in the nucleus of the 2-cell embryo might also be the result of transcription. Our results from RNA FISH and immunocytochemistry analyses positively correlate with previously published results [8,19]. In addition to global RNA found in the nucleus, we detected the partial presence of specific mRNAs *Dazl* and β*-actin* in the nucleoplasm. *Neat2 lncRNA* was present exclusively in the nucleus of the oocyte. In contrast, in the 2–cell embryo, *Neat2* appeared also in the cytoplasm. *Neat2* lncRNA and other RNAs of this class are known to be localized in the nuclei of mouse embryonic fibroblasts and HeLa cells [7,51].

Although findings on translation in the nuclei of cells have been published [68,69], and we detected a number of ribosome components (5.8S rRNA RPS14, RPS3, RPL7) in the nuclei, our other results (absence of phosphorylated RPS6 in the nucleus of the both cell types, and presence of the translational inhibitor 4E-BP1 in the nucleoplasm) indicate that the mRNAs present in the nucleus are translationally dormant [70]. In addition, the translational repressor 4E-BP1 was active in both stages, the oocyte and the embryo (consistently with [19]). The localization of 4E-BP1 in the 2-cell embryo might have a role in the post-transcriptional regulation [71] of the newly synthetized RNA upon embryonic gene activation [72]. The localization of ribosomal components in the nucleoplasm suggests a known mechanism of ribosome biogenesis in the nucleus [73,74]. eIF4A3 is the component of translation initiation complex and also of the exon junction complex, and has a dominant nuclear localization signal sequence [75] which might be responsible for the localization of the protein after the pioneer round of translation [76]. CPEB proteins are key players in the polyadenylation of maternal transcripts [11,20,77] and thus ensure their translation. Our previous work on the mammalian oocyte [21] revealed that CPEB1 was degraded after the resumption of meiosis. Igea and Mendez (2010) proposed that, in the frog oocyte, CPEB4 is accumulated during the second meiotic division and thus can substitute for degraded CPEB1. These authors further suggest that a mechanism is present in *Xenopus* which enables CPEB4 to replace later polyadenylation events in the oocyte. However, we found that CPEB4 was also abundant in the oocyte prior to the resumption of meiosis when CPEB1 was also abundant and functional, suggesting a different mechanism of CPEB regulation in the mammalian oocyte.

We found that selected RBPs, hnRNPA1, eIF4A3, CPEB4 and translational repressor 4E-BP1 were expressed at the same level in the oocyte and in the embryo. However, the ribosomal components RPS14 and phosphorylated RPS6(S235/236) showed significant reduction in the 2-cell embryos. On the other hand, ribosomal components RPS3, RPL7 and RPL24 did not show significant changes in the embryos. We observed different localization of components of ribosomal subunits. During ribosome biogenesis, proteins change their localization, and they also have different localization if they are unbound from ribosomal subunits. We assume that localization of RPS3, RPS14 and RPL7 in the nucleus is necessary for ribosome biogenesis [76, 77, 78, 79] prior to nuclear envelope breakdown to efficiently localize translational machinery to the newly developing spindle [8,9,58].

It is accepted that maternal components (RNA, proteins, organelles) are eliminated after fertilization [32,80]. It is possible that mammalian oocytes and embryos adjust their pool of rRNA and ribosomes to match the mRNA pool and protein requirements, with high levels during oocyte growth, and a relative quiescence in MII stage is accompanied by global reduction of protein synthesis [81–83]. The presence or absence of specific ribosomal proteins in the ribosome is known to control translation of specific subsets of mRNAs [84,85]. This suggests that the elimination of maternal ribosomes might lead to changes in the RNA metabolism between oocyte to embryo transition. Ellederova et al. (2006) and Susor et al. (2008) observed a decrease in global translation during early embryo development, which supports our results generated by PLA showing that 2-cell embryos display a 16% significant decrease in ribosome assembly. In addition, we found that the interaction between RPL24 and RPS6 decreased after the disruption of active ribosomes by puromycin [86], indicating that the interaction foci were active translational sites. We propose that the studied components of the translational machinery are essential for oocyte and embryo development, consistent with [8,19,58]. Monti et al. (2013) discovered that RPL24 and RPS6 are transcribed at the end of the transcriptional activity of the oocyte and are essential for the oocyte to support early embryo development. Ellederova et al. (2006) and Tomek et al. (2002) also observed that despite a decrease in overall protein synthesis in the mammalian oocyte during meiosis, there is a regulatory program that ensures temporal and spatial synthesis of specific proteins that are essential for meiotic progression and embryo development. Similarly with this basic principle we have confirmed the decrease in translation in 2-cell embryos by PLA and HPG methods.

Moreover, we showed in situ translation of the well-known cytoskeletal protein β-actin in the live oocyte. A quantification of the images showed a sevenfold increase of fluorescent intensity in ReAsH and GFP in the patches within the cortex. *In vivo* transcription of the construct DNA showed the translation of β-*actin* RNA in the area of protein localization in the cell.

Although the human oocyte is extremely valuable as the gold standard for assessing clinical relevance, using this cell type is limited in several ways. By starting with the identification of the localization of transcriptome and RBPs in the mouse oocyte and the application of selected markers to the human oocyte, we have found a similar localization of poly(A) RNA and proteins 4E-BP1 and eIF4A3 in both mammalian species, which suggests similar RNA metabolism in human and mouse GV oocytes.

Our findings provide a fundamental insight into the cellular architecture of maternal RNAs in oocytes and embryos from two different mammalian species, mouse and human. We provide a view of the localization of ribosomal proteins, revealing unique and unexpected roles for the translation machinery itself in directing essential aspects of oocyte and early embryo development.

## Supporting information

S1 Fig Controls used in the smRNA FISH. A) Detection of RNA coding GFP in the oocyte. Detection of non-endogenous RNA coding eGFP in the oocyte. Non-endogenous RNA detection was used as a negative control. *GFP* (green) and DAPI (blue). The gray scale shows separated light channels. The white line indicates the cortex of the oocyte and the arrow with the white line indicates the nucleus of the oocyte. The experiment was repeated 3 times, with 15 mouse oocytes per experiment. Scale bars 20 μm. B) As negative controls for smRNA FISH, RNA was digested by RNase A treatment in the fixed oocytes. Oocytes were probed for *Dazl*, β*-actin* and *Neat2* RNAs. The gray scale shows separated light channels: RNA (green) and DNA (blue). The white line indicates the cortex of the oocyte. The arrow with the white line indicates the nucleus of the oocyte. The asterisk indicates cumulus cells. Scale bars 20 μm.
(TIF)

S2 Fig Localization of RBPs in the mouse oocyte after RNA digestion. Treatment with RNase A leads to decrease of signal intensity and disruption of granular structure of 4E-BP1. Single Z-stack from confocal images of mouse GV oocytes and detail of nuclei. We used for 4E-BP1 (green) and eIF4A3 (red) antibodies and stained DNA with DAPI (blue). The gray scale shows different light channels. The experiment was repeated 3 times, with 25 oocytes per experiment. Scale bars represent 10 μm.
(TIF)

S3 Fig Images of whole WBs. Images of WBs probed for specific proteins with depicted molecular size.
(TIF)

S4 Fig Detection of in situ translation in oocyte and embryo. A) Single Z-stack of confocal image shows nascent translation (red) in GV oocytes and 2-cell embryos. Cells were cultured for 30 minutes in the presence of methionine analog HPG and nascent translation was visualized by Click-IT chemistry. The suppression of translation by potent inhibitors puromycin and cycloheximide (CHX) inhibits the incorporation of HPG to the newly synthetized proteins. The white line indicates the cortex of the oocyte. Representative images of at least three independent experiments are shown. DNA stained with DAPI (blue). The gray scale shows light channel for HPG. The arrow indicates the ridge of blastomeres. The asterisk indicates cumulus cells. Scale bars 20μm.
B) Quantification of fluorescence intensity of HPG signal after treatment by puromycin or CHX in GV oocytes and 2-cell stage embryos. The experiment was repeated 3 times, with 25 oocytes per experiment. Data are represented as mean ± s.d.; the values bars with *ns* are not significant, and the asterisk denotes statistically significant differences *p<0.05; **p<0.01; ***p<0.001.
(TIF)

S5 Fig Visualization of actin microfilaments in oocyte and 2-embryo. Single Z-stack of confocal images. Actin filaments visualized by phalloidin (green) and DNA stained with DAPI (blue). Representative images of at least three independent experiments are shown. Scale bars represent 10 μm.
(TIF)
