# Supplementary figures and images for: Localization of RNA and translation in the mammalian oocyte and embryo
Source: PLoS One. 2018 Mar 12;13(3):e0192544. doi: 10.1371/journal.pone.0192544 (PMC5846722; doi:10.1371/journal.pone.0192544)

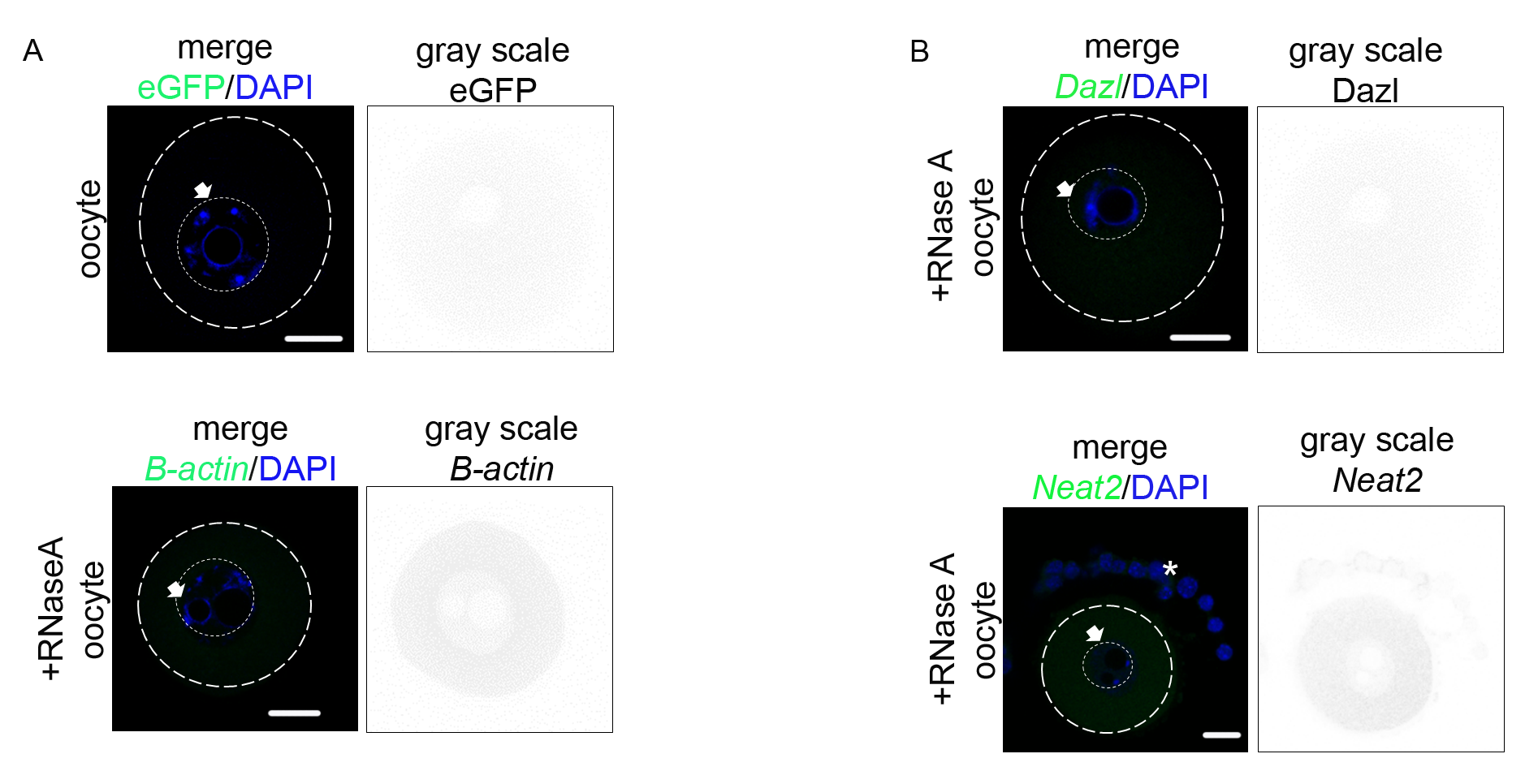

Supplement: S1 Fig — A) Detection of RNA coding GFP in the oocyte. Detection of non-endogenous RNA coding eGFP in the oocyte. Non-endogenous RNA detection was used as a negative control. GFP (green) and DAPI (blue). The gray scale shows separated light channels. The white line indicates the cortex of the oocyte and the arrow with the white line indicates the nucleus of the oocyte. The experiment was repeated 3 times, with 15 mouse oocytes per experiment. Scale bars 20 μm. B) As negative controls for smRNA FISH, RNA was digested by RNase A treatment in the fixed oocytes. Oocytes were probed for Dazl, β-actin and Neat2 RNAs. The gray scale shows separated light channels: RNA (green) and DNA (blue). The white line indicates the cortex of the oocyte. The arrow with the white line indicates the nucleus of the oocyte. The asterisk indicates cumulus cells. Scale bars 20 μm. (TIF) [file pone.0192544.s001.tif]

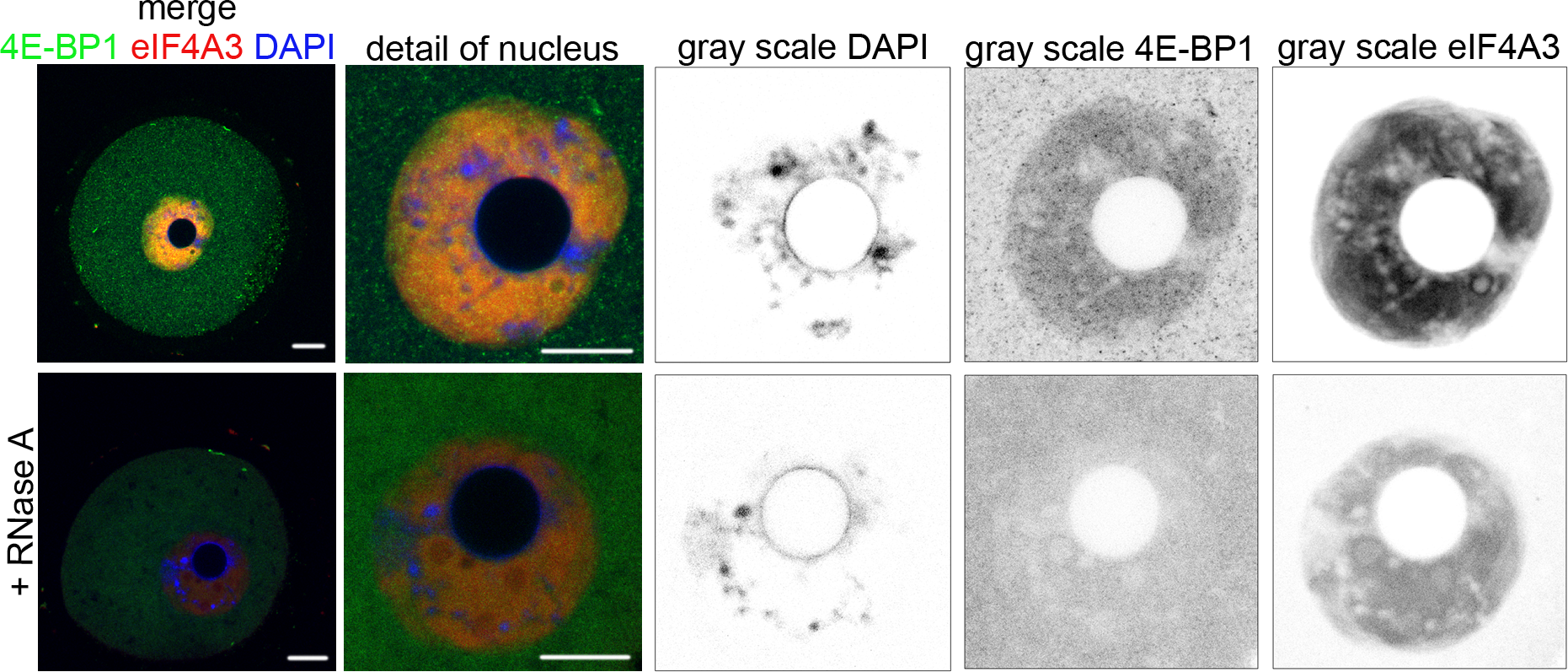

Supplement: S2 Fig — Treatment with RNase A leads to decrease of signal intensity and disruption of granular structure of 4E-BP1. Single Z-stack from confocal images of mouse GV oocytes and detail of nuclei. We used for 4E-BP1 (green) and eIF4A3 (red) antibodies and stained DNA with DAPI (blue). The gray scale shows different light channels. The experiment was repeated 3 times, with 25 oocytes per experiment. Scale bars represent 10 μm. (TIF) [file pone.0192544.s002.tif]

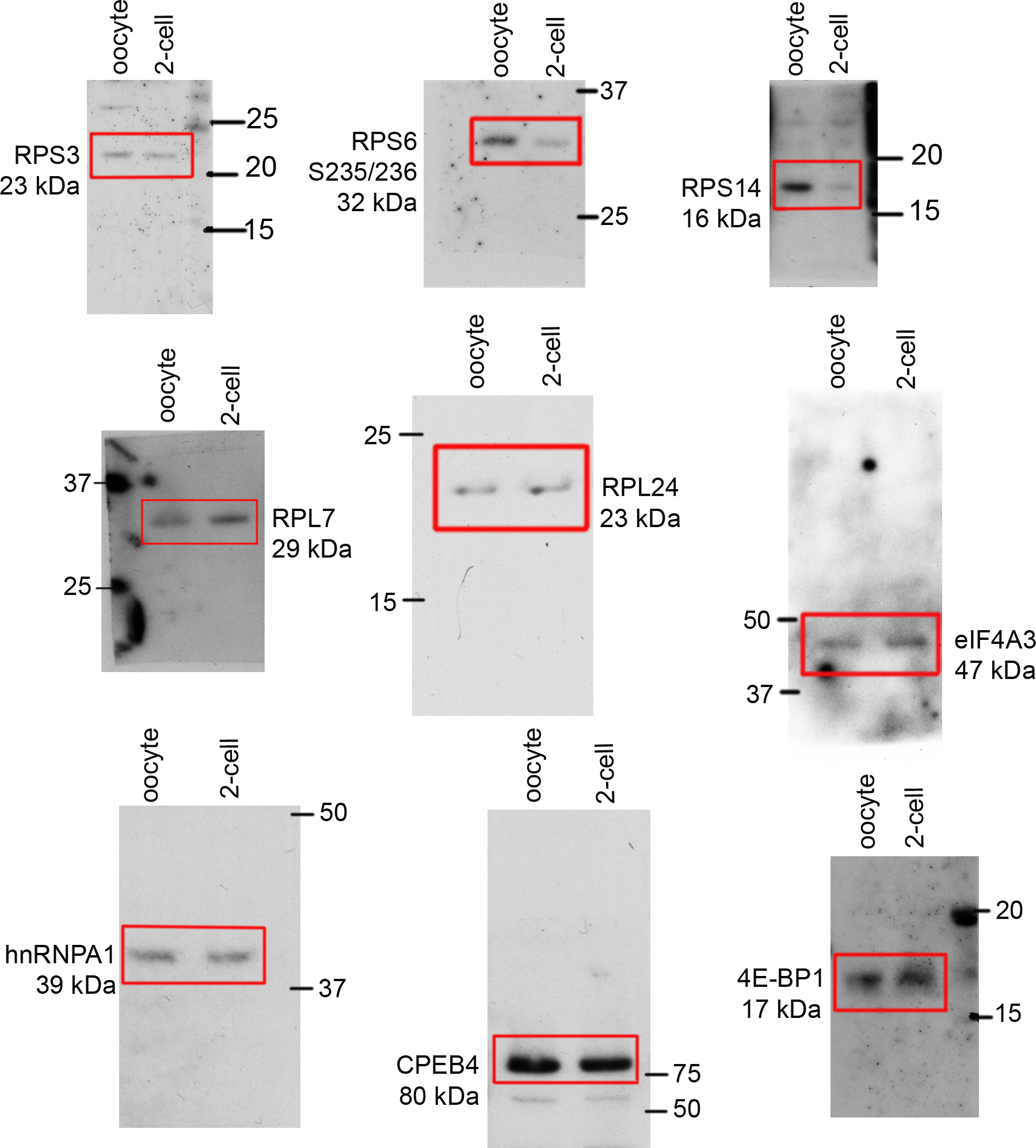

Supplement: S3 Fig — Images of WBs probed for specific proteins with depicted molecular size. (TIF) [file pone.0192544.s003.tif]

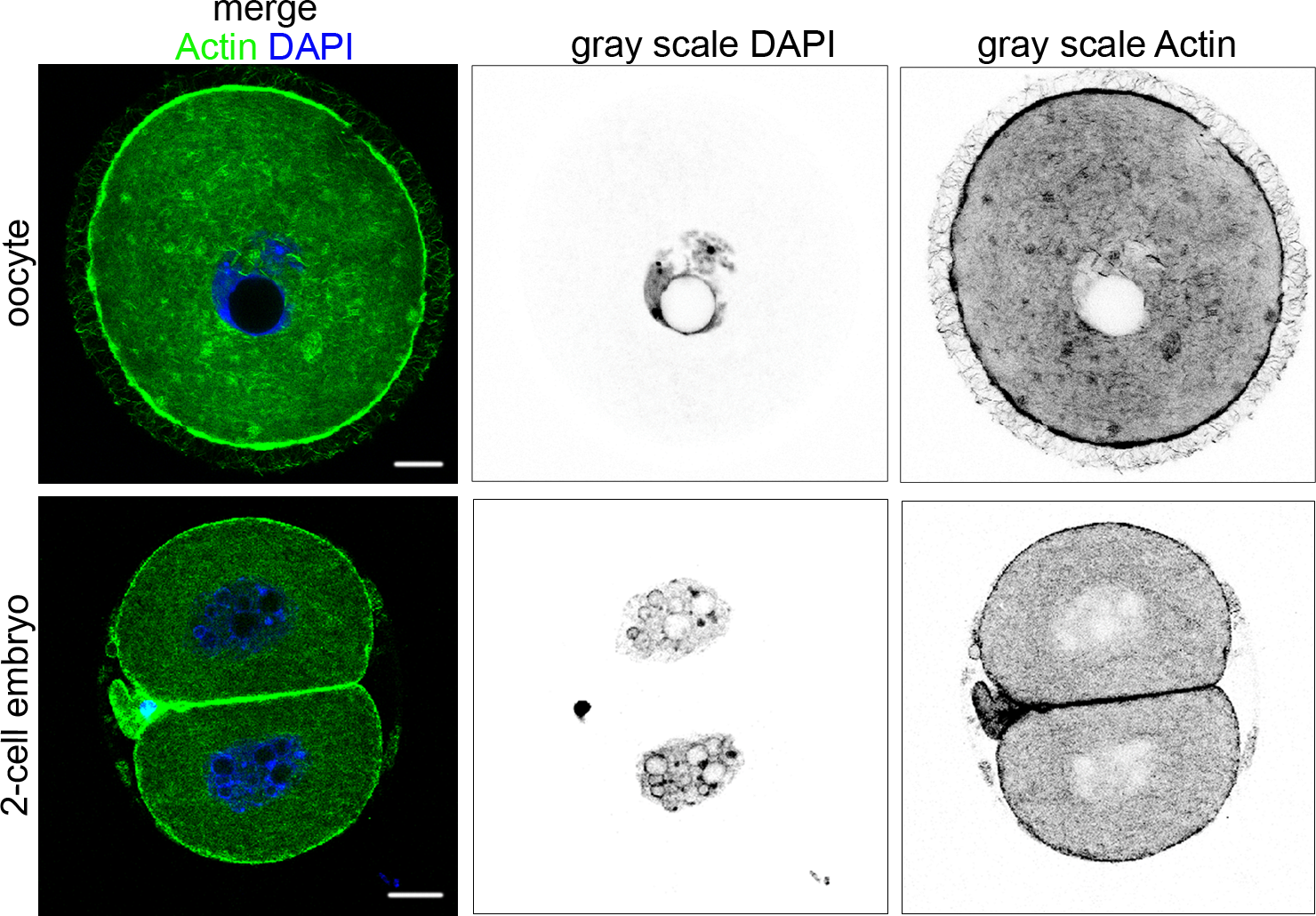

Supplement: S4 Fig — A) Single Z-stack of confocal image shows nascent translation (red) in GV oocytes and 2-cell embryos. Cells were cultured for 30 minutes in the presence of methionine analog HPG and nascent translation was visualized by Click-IT chemistry. The suppression of translation by potent inhibitors puromycin and cycloheximide (CHX) inhibits the incorporation of HPG to the newly synthetized proteins. The white line indicates the cortex of the oocyte. Representative images of at least three independent experiments are shown. DNA stained with DAPI (blue). The gray scale shows light channel for HPG. The arrow indicates the ridge of blastomeres. The asterisk indicates cumulus cells. Scale bars 20μm. B) Quantification of fluorescence intensity of HPG signal after treatment by puromycin or CHX in GV oocytes and 2-cell stage embryos. The experiment was repeated 3 times, with 25 oocytes per experiment. Data are represented as mean ± s.d.; the values bars with ns are not significant, and the asterisk denotes statistically significant differences *p<0.05; **p<0.01; ***p<0.001. (TIF) [file pone.0192544.s004.tif]

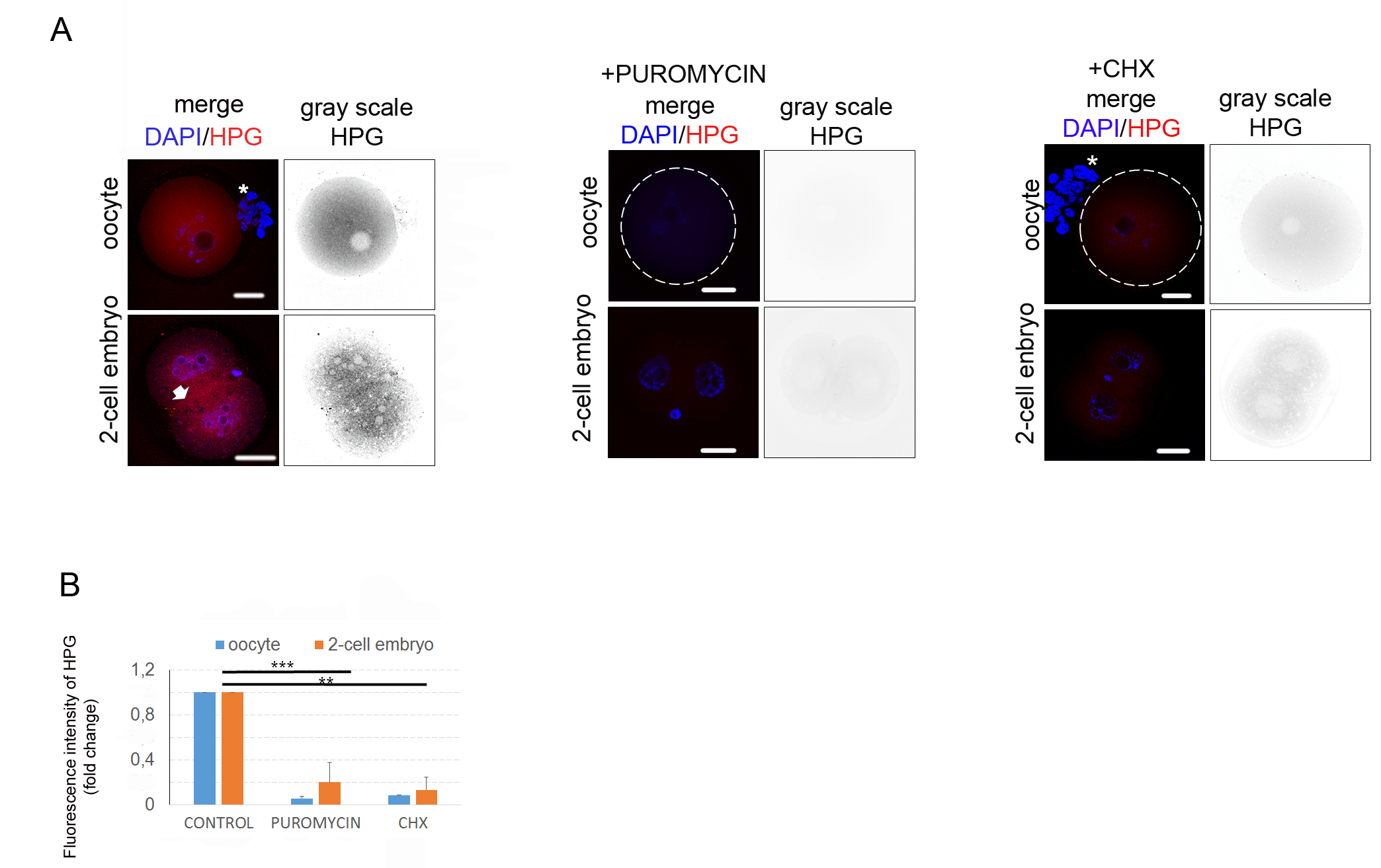

Supplement: S5 Fig — Single Z-stack of confocal images. Actin filaments visualized by phalloidin (green) and DNA stained with DAPI (blue). Representative images of at least three independent experiments are shown. Scale bars represent 10 μm. (TIF) [file pone.0192544.s005.tif]
